# Rapid diagnostics of tuberculosis and drug resistance in the industrialized world: clinical and public health benefits and barriers to implementation

**DOI:** 10.1186/1741-7015-11-190

**Published:** 2013-08-29

**Authors:** Francis Drobniewski, Vladyslav Nikolayevskyy, Horst Maxeiner, Yanina Balabanova, Nicola Casali, Irina Kontsevaya, Olga Ignatyeva

**Affiliations:** 1 Public Health England National Mycobacterium Reference Laboratory, 2 Newark Street, London E1 2AT, UK; 2 Queen Mary University of London, 2 Newark Street, London E1 2AT, UK; 3 University College, University of London, Torrington Place, London WC1E 7HB, UK; 4 Imperial College, London W12 0NN, UK; 5 Samara Oblast TB Service, 154 Novo-Sadovaya Street, Samara, Russian Federation

**Keywords:** Diagnosis, Drug resistance, Tuberculosis, Public health, Whole genome sequencing

## Abstract

In this article, we give an overview of new technologies for the diagnosis of tuberculosis (TB) and drug resistance, consider their advantages over existing methodologies, broad issues of cost, cost-effectiveness and programmatic implementation, and their clinical as well as public health impact, focusing on the industrialized world. Molecular nucleic-acid amplification diagnostic systems have high specificity for TB diagnosis (and rifampicin resistance) but sensitivity for TB detection is more variable. Nevertheless, it is possible to diagnose TB and rifampicin resistance within a day and commercial automated systems make this possible with minimal training. Although studies are limited, these systems appear to be cost-effective. Most of these tools are of value clinically and for public health use. For example, whole genome sequencing of *Mycobacterium tuberculosis* offers a powerful new approach to the identification of drug resistance and to map transmission at a community and population level.

## Introduction

In 2011, 8.7 million people suffered from active tuberculosis (TB) with 1.4 million deaths, with over 95% of these deaths occurring in low- and middle-income countries. TB is also a major killer of those co-infected with human immunodeficiency virus (HIV), causing one quarter of all deaths [1]. TB continues to be a significant public health and clinical problem in the industrialized world.

Within countries of the World Health Organization (WHO) European Region those in the East have much higher notification rates than in the West. The Region reported 309,648 new episodes of TB (34.0 per 100,000 people) with more than 60,000 deaths estimated as being due to TB, or 6.7 cases per 100,000 people [2]. Notification rates for newly-detected and relapsed TB cases in the WHO 18 High Priority Countries (all from the central and eastern part of the European Region), remained almost eight times higher (68.5 per 100,000 people) than in the rest of the Region (8.4 per 100,000) [2].

Combination drug therapy has been the mainstay of TB treatment for decades and six-month short-course rifampicin-based regimens will cure almost all cases. However, interrupted and incomplete therapy selects for drug resistant strains, which are more difficult to treat successfully. Multidrug-resistant tuberculosis (MDR-TB) is caused by bacteria resistant to, at least, isoniazid and rifampicin, two key first-line anti-TB drugs. Although treatable with second-line drugs, therapy is less effective, more toxic and prolonged, requiring up to two years of treatment. Further resistance can develop to form extensively drug-resistant TB, (XDR-TB), that is, MDR-TB strains resistant to any fluroquinolone and amikacin or capreomycin or kanamycin. Strains effectively resistant to all available drugs have emerged.

There were an estimated 310,000 cases of MDR-TB among notified TB patients with pulmonary TB in the world in 2011 with almost two-thirds of the cases occurring in India, China, and the Russian Federation and Former Soviet States, including the Baltic countries [3]. Extensive travel and migration facilitates transmission of resistant strains even to the countries of Western and Central Europe where the rates of drug resistance remain low.

Exciting new advances in TB diagnostics offer the hope of earlier diagnosis, increased cure rates and greater public health benefit by reducing disease transmission. For a long time, laboratories were neglected and considered unimportant in the non-industrialized world with an over-emphasis on the importance of simple microscopy for case diagnosis. In middle- and high-income countries, development continued with innovations in microscopy (for example, light emitting diode (LED) microscopes), microbiological culture (for example, rapid automated liquid culture systems, like the Becton Dickinson MGIT 960 (Becton Dickinson, Sparks, Maryland, USA), nucleic acid amplification systems (for example, Hain Lifesciences (Nehren, Germany) line probe assays and automated systems, such as the Cepheid Xpert® MTB/RIF system (Cepheid, Inc., Sunnyvale, CA, USA).

Although there are point of care (POC) diagnostic tests under development, accurate diagnosis of TB and drug resistant TB requires some form of laboratory infrastructure (ranging from a simple light microscope to molecular diagnostic instruments and/or multi-room laboratory suites to complex biosafety facilities for handling manual and automated liquid culture.

This article gives an overview of new technologies for TB detection as well as drug resistance (including MDR-TB and XDR-TB), focusing on immunocompetent patients in the industrialized world. The role of new diagnostics for TB detection in HIV co-infected individuals and low income countries has been described elsewhere [4-6].

### New microscopy

Light microscopy (LM) of sputa has been the bedrock of TB laboratory diagnosis for decades. It utilizes cheap equipment and materials but is insensitive, non-specific, (especially in the context of industrialized countries where non-TB mycobacterial infections are more common), and requires patient, time-consuming observation of slides. Fluorescent microscopy (FM) is superior in that it is more sensitive than LM, and has a higher throughput, but the equipment and bulbs are expensive [7,8].

Advances in physics led to the development of light emitting diodes (LED), with appropriate fluorescent light output coupled with low power consumption, creating cheaper robust LED FM microscopes, requiring minimal mains or battery power. The WHO has recommended rolling out LED microscopes in lower income settings where they offer the throughput and sensitivity of more expensive fluorescent microscopes and are, therefore, of benefit in high HIV prevalence environments where sputum samples may carry a lower bacterial load; they can also be used successfully in middle or higher income settings [9,10].

For example, a multicenter study assessing the ease and effectiveness of LED-based fluorescence microscopy for TB detection (using PrimoStar iLED (Karl Zeiss, Oberkochen, Germany) was conducted in the Samara Region, Russia in 2008 and 2009 including two sites with no prior experience in fluorescence microscopy (unpublished data). The first phase (“ZN baseline”) aimed to create a control group of Ziel-Nielsen (ZN) stained slides to evaluate false positivity and negativity rates at the demonstration sites. During the validation phase both sites switched to LED-FM after training, followed by implementation where all slides were stained by auramine only. In this Russian study, the overall false positivity and false negativity rates were 5.2% and 1.7%, respectively. The false positive rates for each successive phase were 9.2% (baseline introduction and comparison with current Ziel-Nielsen staining), 4.5% (validation), 1.1% (implementation) and 1.0% (continuation); equivalent false negative rates for each successive phase were 1.7% (baseline and comparison with current Ziel-Nielsen staining), 2.4% (validation), 1.9% (implementation) and 0.9% (continuation). The proportions of false positive and false negative results declined over the stages and the proportion of major errors was almost negligible, demonstrating that LED-FM can be easily implemented in any TB laboratory even with limited prior staff experience. All participating microscopists demonstrated a high level of satisfaction explained by the increased speed of the examination and ease of use.

### Novel molecular amplification test performance for TB diagnosis

Rapid tools for TB detection developed over the last decade in the industrialized world are largely Nucleic Acid Amplification Tests (NAAT) based on amplification of nucleic acids (DNA or RNA), often combined with highly specific detection systems (hybridization with specific oligonucleotide probes or alternatives) to increase sensitivity and specificity of an assay. The polymerase chain reaction (PCR) is the most common methodology utilized in the NAAT; alternatives include real-time PCR (RT-PCR), isothermal, strain displacement or transcription-mediated amplification and ligase chain reaction [11-15] (Table 1).

**Table 1:** Commercially available NAAT assays for TB detection in clinical specimens*

**Assay**	**Manufacturer**	**Method**	**Material**	**Sensitivity,% (95% CI)**	**Specificity,% (95% CI)**	**PLR**	**NLR**	**References**
Amplified MTD	Gen-Probe Inc., San Diego, CA, USA	Transcription-mediated amplification	DNA from decontaminated sputum	86.0 (74.2 to 93.7)	99.3 (96.3 to 100.0)	57.6 (25.5 to 129.9)	0.1 (0.07 to 0.22)	[11,16]
COBAS® TaqMan® MTB Test	Roche Molecular Diagnostics, Pleasanton, CA, USA	RT-PCR	DNA from decontaminated sputum	91.5 (86.9 to 96.1)	98.7 (98.0 to 99.4)	-	-	[17,18]
				79.1	98.2			
*artus*® *M. tuberculosis* PCR	Qiagen, Hilden, Germany	RT-PCR	DNA from decontaminated sputum	97.8 (93.6 to 95.5)	85.1 (75.8 to 91.8)	6.54 (4.0 to 10.8)	0.03 (0.01 to 0.08)	[19]
Loopamp® Tuberculosis Complex Detection Reagent Kit	EIKEN Chemical, Tokyo, Japan	LAMP	Untreated sputum	88.2 (81.4 to 92.7)	-	-	-	[20]
Amplicor MTB	Roche Molecular Diagnostics, Pleasanton, CA, USA	PCR amplification of 16S RNA	DNA from decontaminated sputum	-	-	26.04 (17.04 to 39.80)	0.15 (0.11 to 0.22)	[11]
Cobas Amplicor	Roche Molecular Diagnostics, Pleasanton, CA, USA	PCR amplification of 16S RNA	DNA from decontaminated sputum	-	-	58.59 (37.77 to 90.86)	0.17 (0.13 to 0.22)	[11]
LCx	Abbott Laboratories, USA, Abbott Park, IL, USA	Ligase chain reaction	DNA from decontaminated sputum	88.9 (82.5 to 96.3)	96.8 (95.1 to 98.5)	26.91 (17.21 to 42.09)	0.16 (0.12 to 0.20)	[11,21]
BD Probe Tec Direct	Becton Dickinson, Sparks, MD, USA	Strand Displacement amplification	DNA from decontaminated sputum	77.5 (72.0 to 83.0)	98.0 (97.1 to 98.9)	20.11 (10.42 to 38.82)	0.06 (0.04 to 0.10)	[11,22]
Xpert® MTB/RIF	Cepheid Inc, Sunnyvale, CA, USA	RT- PCR	Smear-positive sputum	98.0 (98.0 to 99.0)	99.0 (99.0 to 99.0)			[13,14,23-28]
Xpert® MTB/RIF	Cepheid Inc, Sunnyvale, CA, USA	RT-PCR	Smear-negative sputum	75.0 (72.0 to 78.0)	99.0 (99.0 to 99.0)			[13,14,23-28]

*Sales of many of these commercial assays have now been discontinued.

NAAT, Nucleic acid amplification techniques; NLR, Negative likelihood ratio; PLR, Positive likelihood ratio.

Speed and improved biosafety are the main advantages of molecular assays: they only require high containment initially and can detect specific nucleotide sequences in processed specimens (crude extracts or treated sputum) within a few hours so the time for the TB detection can be reduced to less than one day.

Although NAAT can theoretically detect a single copy of nucleic acid in a specimen, their sensitivity could be significantly compromised by the presence of PCR inhibitors in clinical specimens and loss of nucleic acids during processing of clinical specimens and, therefore, tends to vary; specificity is usually high (Tables 1 and 2) [11-13,17-20,22]. Recently, line-probe assays (LPAs) and Xpert® MTB/RIF (Cepheid Inc.) have been formally endorsed by the WHO and are now in routine use in many TB laboratories in high- and middle-income countries. Two LPAs currently available on the market for TB detection in clinical specimens (INNO-LiPA Rif.TB (Innogenetics, Zwijndrecht, Belgium) and Genotype® MTBDR*plus* (Hain Lifescience, GmbH, Nehren, Germany) are based on the PCR of specific fragments of the *Mycobacterum tuberculosis* genome followed by hybridization of PCR products to oligonucleotide probes immobilized on membranes.

**Table 2:** Commercially available LPAs for TB detection in clinical specimens

**Assay**	**Manufacturer**	**Method**	**Material**	**Sensitivity,% (95% CI)**	**Specificity,% (95% CI)**	**References**
INNO-LiPA Rif.TB	InnoGenetics, Gent, Belgium	PCR amplification/hybridization	DNA from decontaminated smear-positive sputum	93.0 (92.0 to 94.0)	83.0 (81.0 to 85.0)	[29,30]
INNO-LiPA Rif.TB	InnoGenetics, Gent, Belgium	PCR amplification/hybridization	DNA from decontaminated smear-negative sputum	65.0 (58.0 to 71.0)	96.0 (94.0 to 97.0)	[29-32]
Hain GT MTBDRplus	Hain Lifescience GbmH, Nehren, Germany	PCR amplification/hybridization	DNA from decontaminated sputum	92.0 (90.0 to 94.0)	-	[33,34]

CI, confidence interval; LPA, line-probe assay.

As an example, one large national study in a non-trial context conducted by Seoudi *et al*. [29] examined 7,836 consecutive patient samples over a decade using INNO-LiPA Rif. TB and compared results with a reference standard (conventional liquid and solid media culture with rapid molecular identification and culture-based drug resistance testing). For all sputum specimens (n = 3,382), the sensitivity, specificity, positive predictive value (PPV), negative predictive value (NPV) and accuracy for *M. tuberculosis* complex (MTBC) detection compared to reference microbiology were respectively 93.4%, 85.6%, 92.7%, 86.9% and 90.7%; the equivalent values for smear-positive sputum specimens (n = 2,606) were 94.7%, 80.9%, 93.9%, 83.3% and 91.3%.

Xpert® MTB/RIF is a fully automated RT-PCR-based assay. Much of its increased sensitivity is due to the high volume of sputum that is effectively sampled and compared to other NAAT systems, which is an important lesson to developers of the next generation of tests.

### Diagnosing TB and drug resistance simultaneously

While there have been accurate solid-media-based microbiological tests for drug resistance for decades, the use of commercial (for example, MGIT 960) [35,36] and non- commercial liquid culture systems (for example, microscopic observation drug susceptibility (MODS), Thin Layer Agar (TLA) [37-41] from cultures or sputum have facilitated more rapid diagnosis.

Encouragingly, in May 2009, the 62^nd^ World Health Assembly (WHA62.15) urged member states to take action to achieve universal access to diagnosis and treatment of M/XDR-TB by 2015.

However, real advances in the rapid (less than one to two days) diagnosis of clinically-significant drug resistance have been more recent, requiring identification of mutations in genes responsible for resistance [42-46].

In 1998, an algorithm was proposed for a centralized regional/national service using a combination of novel amplification-based technology for rapidity, coupled with automated liquid culture-based systems for sensitivity of detection and first-line drug susceptibility [47]. The world’s first nationally-available service was established in the UK in 1999.

#### Line probe assays

At that time, in-house and commercial LPAs were available which could detect TB and rifampicin (RMP) resistance (as the overwhelming majority of resistance is caused by mutations in a single gene, *rpoB*, the RNA-dependent DNA polymerase). These assays rely on the PCR-amplification of the region of interest followed by binding to DNA probes bound to a solid membrane; binding is detected colorimetrically, usually as visible bands corresponding to the presence of TB and a sensitive or resistant genotype. Currently, the main commercial LPAs for the rapid diagnosis of TB (INNO-LiPA Rif.TB (Innogenetics, Zwijndrecht, Belgium), GenoType® MTBDR/MTBDRplus and Geno-Type® MTBDR*sl* (both Hain Lifescience), as well as the Xpert® MTB/RIF system mentioned above, are also capable of rapid detection of resistance to rifampicin and (GenoType® MTBDR*plus* only) isoniazid. These tests are designed for use on *M. tuberculosis* isolates and/or primary respiratory specimens [11,29,31,32,48,49].

The GenoType® MTBDR*sl* is the only available rapid assay for detection of resistance to fluorquinolones (FQs), injectable second-line drugs (as well as ethambutol (EMB)) and so offers a rapid detection of XDR-TB in mycobacterial cultures [50,51].

#### Xpert® MTB/RIF

The Xpert® MTB/RIF (Cepheid Inc.) is a fully automated RT-PCR- based assay for the detection of TB bacteria and resistance to RMP in clinical specimens [13,14] and reviewed in [49] (also see Table 1). For TB diagnosis in sputum smear-positive samples, studies showed sensitivities ranging from 93% to 98% and specificities of 83% to 99% [13,14,23-25].

For TB diagnosis, the overall sensitivity of RMP resistance detection in patient specimens for the INNO-LiPA, MTBDRplus and Xpert MTB/RIF assays was, respectively, 93% (95% CI 89 to 96), 97% (95% CI 92 to 99) and 98% (95% CI 97 to 99), for the studies indicated. The pooled specificity of INNO-LiPA, MTBDRplus and Xpert was, respectively, 99% (95% CI 99 to 100), 98% (95% CI 95 to 99) and 99% (95% CI 98 to 99) [11,13,14,23-25,29,31,32,48],[49].

The introduction of Xpert® MTB/RIF-based diagnosis increased TB case findings in India, South Africa and Uganda compared to the use of simple microscopy and clinical diagnosis from 72% to 85% to from 95% to 99% of the cohort of individuals with suspected TB [52].

In the excellent WHO-led global roll-out document for Xpert® MTB/RIF [53], the key programmatic issues for countries with a low prevalence of rifampicin resistance (and MDR) TB is the low PPV of a positive resistant result. This is the situation that applies in most industrialized countries currently and means that most “resistant” isolates will be false-positive ones. This necessitates the use of a second, usually microbiological, test to confirm resistance. The corollary is that the NPV is good.

### Cost of new diagnostics

There have been limited studies on the cost and cost-effectiveness of novel rapid tests and, in particular, the real cost of the entire process (rather than simply the cost of the material) and the overall clinical/diagnostic pathway, which will influence the uptake.

As of 31 December 2012, a total of 966 Xpert® MTB/RIF instruments (5,017 modules) and 1,891,970 MTB/RIF cartridges had been purchased in the public sector in 77 of the 145 countries eligible for concessionary pricing [54].

Pantoja *et al*. recently [55] assessed the cost, globally and in 36 high-burden countries, of two strategies for diagnosing TB and multidrug-resistant TB (MDR-TB): Xpert® MTB/RIF with follow-on diagnostics, and conventional diagnostics. They showed that using Xpert® MTB/RIF to diagnose MDR-TB would cost US$0.09 billion/year globally and be of lower cost than conventional diagnostics globally and in all high TB burden countries (HBCs). Diagnosing TB in HIV-positive people using Xpert® MTB/RIF would also cost about US$0.10 billion/year and be of lower cost than conventional diagnostics globally and in 33 of 36 HBCs.

Testing everyone with TB signs and symptoms would cost almost US$0.47 billion/year globally, much more than conventional diagnostics. However, in European countries, Brazil and South Africa the cost would represent <10% of overall TB funding. The authors concluded that while using it to test everyone with TB signs and symptoms would be affordable in several middle-income countries, the financial viability in low-income countries would require large increases in TB funding and/or further price reductions.

Kirwan and colleagues [56] argued that studies on Xpert® MTB/RIF have shown cost-effectiveness in some but not all settings. They pointed out that serial implementation of new technologies can cause ineffective spending and fragmentation of services. The process by which new tests are incorporated into existing diagnostic algorithms would affect both outcomes and costs. They argue that more detailed data on performance, patient-important outcomes and costs when used with adjunct tests were needed for each setting before implementation and that while awaiting further clarification it would seem prudent to slow implementation among resource-constrained tuberculosis control programs [56].

Vassall *et al*. [52] showed that when Xpert® MTB/RIF was used as a screening tool for testing all TB suspects in India, South Africa or Uganda, the cost and cost-effectiveness increased from US$28 to US$49 from US$133 to US$146, and US$137 to US$151 per TB case detected if Xpert® MTB/RIF is used “in addition to” and “as a replacement of” smear microscopy.

Calculated incremental cost effectiveness ratios (ICERs) for using Xpert® MTB/RIF “in addition to” smear microscopy ranged from US$41 to $110 per disability adjusted life year (DALY) averted and were below the WHO threshold and, therefore, indicate Xpert® MTB/RIF to be a cost-effective method of TB diagnosis in low- and middle-income settings. However, scale and range of current TB diagnostic algorithm practice in other settings would determine the extent of the cost-effectiveness of adding this new tool into routine practice [52].

Conversely, in high income countries the diagnostic yield and cost-effectiveness will differ as microbiological culture and/or DST will form the base case scenario. Rapid diagnostics may have even greater financial impact in identifying those patients with risk factors for MDR-TB but who subsequently were shown to have simple drug sensitive TB, that is, the shorter cost of enhanced isolation facilities within institutions may justify the increased diagnostic cost. For example, in one UK London teaching hospital the use of line probe assays would have created potential annual savings of between £50,000 and £150,000 a decade ago [57].

There are no complete costs for the diagnosis and management of TB which also include societal costs. A review in 2007 calculated a UK annual drug bill of £1.95 million/year (2002 costs) [36]. The mean costs (including in- and out-patient stay, drugs, toxicity monitoring) of managing drug sensitive and MDR-TB cases in London, UK were estimated to be approximately £6,000 and £60,000 in a study from 2000 [58]. In a more recent German study, the costs were comparable with a mean combined in-patient and out-patient costs of €7,364 and to €52,259 for the treatment of a drug sensitive and MDR-TB cases, respectively [59].

This is in broad agreement with a WHO report which showed that the drug cost for treating drug-resistant patients was approximately 50 to 200 times higher than for treating a drug-susceptible TB patient, with the overall costs of care at least 10 times higher [3]. However, the overall societal costs are more difficult to measure. In the recent German study [59], 4,444 new cases of TB were reported in 2009, with 2.1% (63 cases) MDR-TB cases. The mean costs of treatment per TB case overall, including treatment of MDR-TB, was €7,931 to which was added the mean cost due to loss of productivity (€2,313), costs per case for rehabilitation (€74) and contact tracing (€922), giving a total of €11,240 as the overall societal cost. In a report from 2012 [60], the UK reported 8,917 cases and 60 MDR-TB cases in 2009. As the UK and German TB case management approaches are broadly similar if we use the same societal cost figure, then UK societal costs for TB are approximately €100 million. The equivalent mean treatment cost would be a little over €70 million.

There is a real need to model and cost end-to-end services rather than perform simple analyses around the cost of the diagnostic alone. It may be more cost effective in a high income, high cost environment to control the whole process carefully for quality and to adjust workflow. For example, the cost and cost-effectiveness of the entire process in the UK will be influenced by poor transport logistics. Alternative service delivery models involving the leasing of vehicles with mobile phlebotomist-technicians, for a blood sample-based test, who can either bring samples to the laboratory rapidly for analysis, or perform a POC test on site may be more cost-effective than the current practice.

### Barriers to uptake

There has been a relatively slow uptake of new TB diagnostics, some of which have been available since the 1990s [47].

Within the UK health model, greater laboratory costs should be offset against increased hospital (institutional) savings to encourage innovation and reduce barriers to adoption of newer tests by demonstrating rational cost savings in place of simplistic percentage cost cuts used currently. Other insurance-based health models, such as those in France or Germany, are arguably better at implementing proven diagnostics.

The underlying tension existing for all diagnostic tests continues to be the debate over the merits of point of care tests versus those performed in a more centralized laboratory environment. Assuming the technical issues are solved, arguably the greatest influence on whether it is more cost-effective to bring samples to a laboratory or use point of care tests, depends on transport logistics.

### Public health relevance and impact of new diagnostics

#### Active TB

Any diagnostic tool may be of value clinically, for public health or both. Clinically, we value a reduction in mortality and modeling suggests a 100% sensitive and specific test with 100% access could prevent up to 36% of TB related death [61]. Other models estimate that employing more sensitive and rapid tests would produce between a 17% and 23% mortality reduction [62,63].

However, a patient who survives but remains infectious with TB, especially highly drug resistant TB, may be of greater importance and danger from a public health perspective. Globally, introduction of new diagnostics without anticipation and planning for an increase in the number of cases diagnosed could lead to disaster at the programmatic level as more patients are placed on TB therapy, which then runs out; incomplete therapy remains the overriding cause of clinically relevant drug resistance [64-66]. Highly industrialized countries have the financial ability to increase expenditure to compensate for this increased treatment requirement at a programmatic level but there remains a need to understand this need and plan for it.

Equally, diagnostic delay has led to failures in adopting appropriate public health measures and has been documented in many high-income jurisdictions, for example, in New York [67]. Patient and health service delays were identified in a retrospective cohort study of patients with pulmonary tuberculosis notified between 1 April 2001 and 1 March 2002 in London, UK. The median case finding delays were between 78 and 99 days. The median patient-related delay was between 34.5 and 54 days and the median health care-related delay was 29.5 days. Shorter case finding delays were found in patients born in a high prevalence country, patients presenting first to an Accident and Emergency department (A&E), with limitations in TB service capacity and organizational factors accounting for much of the delay [68].

These points and the potential effects of new versus old procedures on public health efforts are summarized in Table 3 and some of these are discussed in more detail below. Clearly a rapid, highly specific and sensitive, active TB test would be of equal clinical and public health value. However, sputum smear microscopy has been criticized because it is too insensitive and not specific enough for TB. Nevertheless, because of its relative insensitivity, it is a good test of infectivity, identifying those individuals with the highest bacterial load who are, therefore, the most infectious and a priority for public health intervention. Therefore, the urgent midnight sputum smear examination may be of less clinical diagnostic benefit but is essential to prioritize institutional and community isolation procedures. A new POC diagnostic test for TB, for example, with excellent specificity for MTBC, but similar sensitivity to smear microscopy, may be of limited clinical value but excellent public health value helping to identify priority infectious cases and limit transmission of TB further. For example, a study in South Africa attempted to use the cycle threshold values of the Xpert system as a rule-in/rule-out test for smear positivity and so infectivity; it had poor value as a rule-in test but moderate value as a rule-out, although 20% of individuals would have been erroneously ruled out as smear negative [69].

**Table 3:** The impact of new diagnostic tools for TB on the public health system

**Initial versus new tool**	**Use**	**Staffing impact/advantage**	**Patient impact/advantage**	**Laboratory advantage**	**Reading**	**Public health impact of second tool**
TST versus gamma interferon release	Latent TB	Qualified nurse to apply and read TST vs phlebotomist	Two versus one visit	Low versus higher specificity	Moderately standardized versus more precise cut-off	Fewer referrals due to more specific diagnosis LTBI.
	infection (LTBI)					
Solid versus liquid culture	Active TB	Unchanged	Improved sensitivity	Higher sensitivity for detection (but offset if higher contamination)	Non-automated versus automated cut-off	Identification of all TB cases reduce transmission
Smear* versus Xpert® MTB/RIF system or LPA	Active TB	Arguably less trained staff needed	Greater sensitivity; but no indicator of infectivity	Low versus high sensitivity and specificity	Variable versus cut-off	Fewer false positive results
		Due to inactivation lower risk of staff infection				
Smear *versus Xpert® MTB/RIF system or LPA, for example, GenoType® MDRTB*Plus*	Drug resistance	Less qualified personnel initially for interpretation	Short turnaround time for marker antibiotic	No versus one key marker antibiotic (rifampicin) and also isoniazid for LPA	Variable versus exact cut-off	Immediate availability of marker antibiotic results; poor PPV in low prevalence areas
Phenotypic versus GenoType® MTBDR*sl* line probe assay (LPA) for FQ, injectable agents	Drug resistance	Unchanged for qualification of staff, reduced risk for staff infection	In areas with high MDR rates shorter turnaround for XDR-TB detection	Earlier XDR-TB screen and set-up of other drug testing for treatment	Simpler cut-off; limited drug range	Immediate preliminary screening for MDR- and XDR-TB and aid planning contact investigation

* “Smear” means the microscopic examination of sputum.

LPA, line prove assay; PPV, positive predictive value; MDR-TB, Multidrug-resistant tuberculosis; TST, tuberculin skin testing; XDR-TB, extensively drug-resistant TB; LTBI, Latent TB infection.

An assay for drug resistance (for rifampicin, isoniazid and MDR-TB) would be equally valuable for clinical management and public health; correct therapy helps the patients and, by rendering the individual non-infectious, reduces disease transmission. However, there is a further dimension in that by establishing the correct level of isolation, disease transmission will be interrupted. Tests for second line drug therapy, for example, to establish XDR-TB, are arguably of greater clinical value in that the level of isolation and concern would have been established by the MDR-TB diagnosis.

Such improvements in the speed and/or sensitivity of diagnosis of TB and drug resistance have greatest potential impact on the clinical management of those co-infected with HIV due to the high mortality associated with MDRTB/XDRTB in middle or high income countries [70-74].

#### Latent TB infection (LTBI)

Although this review is focused on active TB, several industrialized countries are entering a phase of (potential) TB eradication in their TB programs; in these states new cases will come from latently infected migrants and the indigenous population as it ages. Better identification of truly latently-infected individuals offers the opportunity to interrupt the onset of active (infectious) TB. Blood tests based on Gamma-interferon release (IGRA) provide an improvement in specificity on classical tuberculin skin testing (TST) as they are not influenced by prior Bacillus Calmette–Guérin (BCG) vaccination [75-77].

Nevertheless, the value of IGRA tests clinically differs from their public health value and both are dependent on the willingness of well-feeling individuals to accept and complete TB chemoprophylaxis, which is challenging - both have value in identifying a significant exposure in a contact investigation and in preventing further harm, but they can also potentially provide wider programmatic understanding of undiagnosed TB transmission in a community. For example, in Baltimore, IGRA implementation of LTBI evaluation in a public health clinic significantly reduced the proportion of referred individuals with preliminary suspicion of LTBI diagnosis in whom LTBI was finally diagnosed, but IGRA testing had no impact on treatment initiation or completion [75].

#### Molecular epidemiological typing and Next Generation Sequencing

This is a good example of new “diagnostic” techniques, which are primarily of public health importance, establishing unknown routes of disease transmission, and confirming or refuting institutional outbreaks. Arguably, the clinical value of these techniques is in primarily identifying weaknesses in institutional infection control procedures which lead to new TB cases and in establishing the value of TB programmatic changes in reducing transmission. For example, in a city, region or country improvements in TB control through early diagnosis, effective treatment and improved infection, control may be masked by new migration of TB cases. Techniques, such as variable number tandem repeat (VNTR) [78] and next generation sequencing, may [79] all be of value in establishing improvement by showing that fewer TB cases were clustered together (clustering indicating TB transmission between individuals). Reduced rates of TB case clustering together with data indicating cure rates over 85% and reduced rates of drug resistance development in cases which were initially drug sensitive, provide a portfolio of indicators demonstrating an effective TB program. In San Francisco, for example, DNA fingerprinting showed a reduction in all TB cases and clustered TB cases demonstrating an improvement in TB control [80-82].

Using whole genome sequencing and analyzing genetic distance between isolates from pairs of household contacts in the UK, Walker *et al*. [83] deduced that isolates separated by less than five SNPs (single nucleotide polymorphisms) were likely to be the result of recent transmission events and that transmission could be ruled out if isolates were separated by more than 12 SNPs. In a similar study in The Netherlands, a low TB prevalence country with robust procedures for contact tracing, 97 pairs of epidemiologically-linked isolates differed by an average of 3.4 SNPs [84]. However, no epidemiological link could be established between 82 pairs of isolates that had no SNP differences.

Within a large, population sequencing study in the Samara region of Russia, we established linkages between household contacts but for many clusters of TB strains with no SNP differences, patients lived in geographically-distant parts of the region making direct transmission unlikely (data not shown). While cryptic outbreaks may be uncovered in a low incidence setting [83], the degree of relatedness between unlinked isolates in high prevalence regions may make the establishment of epidemiologic links between patients problematic. Illustrating this, we found that when we applied whole-genome sequencing to TB isolates from Estonia, collected in 2008, they differed by only 16 SNPs from that of a Lithuanian-born patient isolated in the UK in 2011, and within the Beijing clade A in Samara, Russia, the genetic distance between isolates belonging to a Latvian and Russian patient was 13 SNPs. (data not shown). None of these individuals could have been in direct contact. However, a direct transmission link may be ruled out based on a significant genetic distance between isolates.

Furthermore, in low TB prevalence countries, where one migrant population dominates a putative outbreak, an understanding of the phylogeny of *M. tuberculosis* in the patients’ country of origin may be critical to correctly interpret genetic distances and surmise transmission chains. Whole genome sequencing of *M. tuberculosis* can offer a powerful new approach to the identification of drug resistance and to map transmission at a community and population level when carefully interpreted.

## Conclusions

Rapid diagnostics for TB and drug resistance have undergone extraordinary development over the last decade with a major clinical impact on improving TB diagnosis and the early identification of drug resistant TB. These improvements have led to more rapid implementation of the best therapy for given patients. There remains a need for new diagnostics to improve the sensitivity of detection for active TB in children, HIV co-infected patients and extrapulmonary disease. The ideal position should be akin to malaria diagnosis where therapy is no longer automatically given when novel diagnostic tests are negative (ruled-out).

Multiple studies in both the industrialized and non-industrialized world showed that early identification of MDR-TB and the institution of therapy based on susceptibility in laboratory drug-resistance assays led to improved survival. The early and more accurate identification of TB cases, drug resistance and institution of appropriate therapy also removes sources of TB transmission by curing them. This combination of rapid accurate diagnosis and correct treatment is the root of all successful TB programs and public health strategies. Reducing diagnostic delay remains a high clinical and public health priority.

## Abbreviations

BCG: Bacillus Calmette–Guérin; DALY: Disability adjusted life year; EMB: Ethambutol; FM: Fluorescent microscopy; FQ: Fluoroquinolone; HBC: High burden country; HIV: Human immunodeficiency virus; ICER: Incremental cost effectiveness ratio; IGRA: Interferon-gamma release assay; LED: Light-emitting diode; LM: Light microscopy; LPA: Line probe assay; LTBI: Latent tuberculosis infection; MDR-TB: Multidrug-resistant tuberculosis; MODS: Microscopic observation drug susceptibility assay; MTBC: *Mycobacterium tuberculosis* complex; NAAT: Nucleic acid amplification test; NPV: Negative predictive value; PCR: Polymerase chain reaction; POC: Point-of-care; PPV: Positive predictive value; RMP: Rifampicin; RT-PCR: Real-time polymerase chain reaction; SNP: Single nucleotide polymorphism; TB: Tuberculosis; TLA: Thin layer agar assay; TST: Tuberculin skin test; VNTR: Variable number tandem repeats; WHO: World Health Organization; XDR-TB: mxtensively drug-resistant tuberculosis; ZN: Ziehl-Neelsen staining.

## Competing interests

None of the authors declare any competing interests.

## Authors’ contributions

FD prepared the initial draft. VN, HM, YB, NC, IK and OI contributed to the final drafting, and all read and approved the final version.

## Authors’ information

FD is a microbiologist, immunologist and TB physician, Professor of Global Health at Imperial College and Professor of International Health at Queen Mary and an honorary professor, University College, London. He is Director of the UK PHE National Mycobacterial Reference Laboratory and Consultant medical microbiologist and TB physician at Barts Health Trust, London. VN is a microbiologist and molecular biologist with an extensive expertise in development and validation of novel diagnostic assays, molecular epidemiology and immunology. HM is a Consultant medical microbiologist. YB is a TB physician and epidemiologist with extensive experience in implementation and management of multi-center’ studies and data analysis. IK and OI are microbiologists and molecular biologists involved in diagnostic and clinical field studies implementation, coordination and data analysis. NC is a molecular biologist and microbiologist involved in the analysis of DNA sequence data.

## References

[B1] World Health Organization WHO Global Tuberculosis Report 2012. WHO document WHO/HTM/TB/2012.6 2012 Geneva, Switzerland: WHO

[B2] European Centre for Disease Prevention and Control (ECDC), WHO Regional Office for Europe (WHO/Europe) Tuberculosis Surveillance and Monitoring in Europe 2012 Stockholm, Sweden: ECDC/WHO Europe

[B3] World Health Organization Multidrug and Extensively Drug-Resistant TB (M/XDR-TB). Global Report on Surveillance and Response 2010 Geneva, Switzerland: WHO

[B4] Perkins MD Cunningham J Facing the crisis: improving the diagnosis of tuberculosis in the HIV era J Infect Dis 2007 196 S15 27 17624822 10.1086/518656

[B5] Lawn SD Diagnosis of pulmonary tuberculosis Curr Opin Pulm Med 2013 19 280 288 23528956 10.1097/MCP.0b013e32835f1b70

[B6] Chegou NN Hoek KG Kriel M Warren RM Victor TC Walzl G Tuberculosis assays: past, present and future Expert review of anti-infective therapy 2011 9 457 469 21504402 10.1586/eri.11.23

[B7] World Health Organization Fluorescent Light-Emitting Diode Microscopy for Diagnosis of Tuberculosis. WHO Policy Statement. WHO/HTM/TB/2011.8 2010 Geneva, Switzerland: WHO 23586120

[B8] Anthony RM Kolk AH Kuijper S Klatser PR Light emitting diodes for auramine O fluorescence microscopic screening of *Mycobacterium tuberculosis* Int J Tuberc Lung Dis 2006 10 1060 1062 16964802

[B9] Minion J Pai M Ramsay A Menzies D Greenaway C Comparison of LED and conventional fluorescence microscopy for detection of acid fast bacilli in a low-incidence setting PLoS One 2011 6 e22495 21811622 10.1371/journal.pone.0022495 PMC3141065

[B10] Turnbull ER Kaunda K Harris JB Kapata N Muvwimi MW Kruuner A Henostroza G Reid SE An evaluation of the performance and acceptability of three LED fluorescent microscopes in Zambia: lessons learnt for scale-up PLoS One 2011 6 e27125 22073271 10.1371/journal.pone.0027125 PMC3208552

[B11] Ling DI Flores LL Riley LW Pai M Commercial nucleic-acid amplification tests for diagnosis of pulmonary tuberculosis in respiratory specimens: meta-analysis and meta-regression PLoS ONE 2008 3 e1536 18253484 10.1371/journal.pone.0001536 PMC2212137

[B12] Nagdev KJ Kashyap RS Parida MM Kapgate RC Purohit HJ Taori GM Daginawala HF Loop-mediated isothermal amplification for rapid and reliable diagnosis of tuberculous meningitis J Clin Microbiol 2011 49 1861 1865 21411583 10.1128/JCM.00824-10 PMC3122663

[B13] Boehme CC Nabeta P Hillemann D Nicol MP Shenai S Krapp F Allen J Tahirli R Blakemore R Rustomjee R Milovic A Jones M O’Brien SM Persing DH Ruesch-Gerdes S Gotuzzo E Rodrigues C Alland D Perkins MD Rapid molecular detection of tuberculosis and rifampin resistance N Engl J Med 2010 363 1005 1015 20825313 10.1056/NEJMoa0907847 PMC2947799

[B14] Boehme CC Nicol MP Nabeta P Michael JS Gotuzzo E Tahirli R Gler MT Blakemore R Worodria W Gray C Huang L Caceres T Mehdiyev R Raymond L Whitelaw A Sagadevan K Alexander H Albert H Cobelens F Cox H Alland D Perkins MD Feasibility, diagnostic accuracy, and effectiveness of decentralised use of the Xpert MTB/RIF test for diagnosis of tuberculosis and multidrug resistance: a multicentre implementation study Lancet 2011 377 1495 1505 21507477 10.1016/S0140-6736(11)60438-8 PMC3085933

[B15] Pietzka AT Indra A Stoger A Zeinzinger J Konrad M Hasenberger P Allerberger F Ruppitsch W Rapid identification of multidrug-resistant *Mycobacterium tuberculosis* isolates by rpoB gene scanning using high-resolution melting curve PCR analysis J Antimicrob Chemother 2009 63 1121 1127 19369271 10.1093/jac/dkp124

[B16] Amplified Mycobacterium Tuberculosis Direct Test. Package Insert San Diego, CA, USA: Gen-Probe

[B17] Kim JH Kim YJ Ki CS Kim JY Lee NY Evaluation of Cobas TaqMan MTB PCR for detection of *Mycobacterium tuberculosis* J Clin Microbiol 2011 49 173 176 21048015 10.1128/JCM.00694-10 PMC3020466

[B18] Yang YC Lu PL Huang SC Jenh YS Jou R Chang TC Evaluation of the Cobas TaqMan MTB test for direct detection of *Mycobacterium tuberculosis* complex in respiratory specimens J Clin Microbiol 2011 49 797 801 21177901 10.1128/JCM.01839-10 PMC3067742

[B19] Hur M Moon HW Yun YM Kang TY Kim HS Lee KM Kang SH Lee EH Detection of tuberculosis using artus *M. tuberculosis* PCR Kit and COBAS AMPLICOR *Mycobacterium tuberculosis* Test Int J Tuberc Lung Dis 2011 15 795 798 21575301 10.5588/ijtld.10.0367

[B20] Mitarai S Okumura M Toyota E Yoshiyama T Aono A Sejimo A Azuma Y Sugahara K Nagasawa T Nagayama N Yamane A Yano R Kokuto H Morimoto K Ueyama M Kubota M Yi R Ogata H Kudoh S Mori T Evaluation of a simple loop-mediated isothermal amplification test kit for the diagnosis of tuberculosis Int J Tuberc Lung Dis 2011 15 1211 1217 21943848 10.5588/ijtld.10.0629

[B21] Ribeiro FK Dettoni Vdo V Peres RL Vinhas SA Có TR Dietze R Palaci M Evaluation of a commercial test based on ligase chain reaction for direct detection of *Mycobacterium tuberculosis* in respiratory specimens Rev Soc Bras Med Trop 2004 37 431 435 15765589 10.1590/s0037-86822004000600001

[B22] Wang JY Lee LN Hsu HL Hsueh PR Luh KT Performance assessment of the DR. MTBC Screen assay and the BD ProbeTec ET system for direct detection of *Mycobacterium tuberculosis* in respiratory specimens J Clin Microbiol 2006 44 716 719 16517844 10.1128/JCM.44.3.716-719.2006 PMC1393081

[B23] Armand S Vanhuls P Delcroix G Courcol R Lemaitre N Comparison of the Xpert MTB/RIF test with an IS6110-TaqMan real-time PCR assay for direct detection of *Mycobacterium tuberculosis* in respiratory and nonrespiratory specimens J Clin Microbiol 2011 49 1772 1776 21411592 10.1128/JCM.02157-10 PMC3122654

[B24] Marlowe EM Novak-Weekley SM Cumpio J Sharp SE Momeny MA Babst A Carlson JS Kawamura M Pandori M Evaluation of the Cepheid Xpert MTB/RIF assay for direct detection of *Mycobacterium tuberculosis* complex in respiratory specimens J Clin Microbiol 2011 49 1621 1623 21289151 10.1128/JCM.02214-10 PMC3122817

[B25] Ioannidis P Papaventsis D Karabela S Nikolaou S Panagi M Raftopoulou E Konstantinidou E Marinou I Kanavaki S Cepheid GeneXpert MTB/RIF assay for *Mycobacterium tuberculosis* detection and rifampin resistance identification in patients with substantial clinical indications of tuberculosis and smear-negative microscopy results J Clin Microbiol 2011 49 3068 3070 21677069 10.1128/JCM.00718-11 PMC3147726

[B26] Helb D Jones M Story E Boehme C Wallace E Ho K Kop J Owens MR Rodgers R Banada P Safi H Blakemore R Lan NT Jones-López EC Levi M Burday M Ayakaka I Mugerwa RD McMillan B Winn-Deen E Christel L Dailey P Perkins MD Persing DH Alland D Rapid detection of *Mycobacterium tuberculosis* and rifampin resistance by use of on-demand, near-patient technology J Clin Microbiol 2010 48 229 237 19864480 10.1128/JCM.01463-09 PMC2812290

[B27] Moure R Munoz L Torres M Santin M Martin R Alcaide F Rapid detection of *Mycobacterium tuberculosis* complex and rifampin resistance in smear-negative clinical samples by use of an integrated real-time PCR method J Clin Microbiol 2011 49 1137 1139 21191053 10.1128/JCM.01831-10 PMC3067676

[B28] Malbruny B Le Marrec G Courageux K Leclercq R Cattoir V Rapid and efficient detection of *Mycobacterium tuberculosis* in respiratory and non-respiratory samples Int J Tuberc Lung Dis 2011 15 553 555 21396219 10.5588/ijtld.10.0497

[B29] Seoudi N Mitchell SL Brown TJ Dashti F Amin AK Drobniewski FA Rapid molecular detection of tuberculosis and rifampicin drug resistance: retrospective analysis of a national UK molecular service over the last decade Thorax 2012 67 361 367 22213739 10.1136/thoraxjnl-2011-200610

[B30] Ogwang S Asiimwe BB Traore H Mumbowa F Okwera A Eisenach KD Kayes S Jones-Lopez EC McNerney R Worodria W Ayakaka I Mugerwa RD Smith PG Ellner J Joloba ML Comparison of rapid tests for detection of rifampicin-resistant *Mycobacterium tuberculosis* in Kampala Uganda. BMC Infect Dis 2009 9 139 10.1186/1471-2334-9-139 PMC2744678 19709423

[B31] Sam IC Drobniewski F More P Kemp M Brown T *Mycobacterium tuberculosis* and rifampin resistance, United Kingdom Emerg Infect Dis 2006 12 752 759 16704831 10.3201/eid1205.041339 PMC3374436

[B32] Viveiros M Leandro C Rodrigues L Almeida J Bettencourt R Couto I Carrilho L Diogo J Fonseca A Lito L Lopes J Pacheco T Pessanha M Quirim J Sancho L Salfinger M Amaral L Direct application of the INNO-LiPA Rif.TB line-probe assay for rapid identification of *Mycobacterium tuberculosis* complex strains and detection of rifampin resistance in 360 smear-positive respiratory specimens from an area of high incidence of multidrug-resistant tuberculosis J Clin Microbiol 2005 43 4880 4884 16145166 10.1128/JCM.43.9.4880-4884.2005 PMC1234138

[B33] Mironova S Pimkina E Kontsevaya I Nikolayevskyy V Balabanova Y Skenders G Kummik T Drobniewski F Performance of the GenoType((R)) MTBDRPlus assay in routine settings: a multicenter study Eur J Clin Microbiol Infect Dis 2012 31 1381 1387 22037774 10.1007/s10096-011-1453-1

[B34] Nikolayevskyy V Balabanova Y Simak T Malomanova N Fedorin I Drobniewski F Performance of the Genotype MTBDRPlus assay in the diagnosis of tuberculosis and drug resistance in Samara, Russian Federation BMC Clin Pathol 2009 9 2 19284561 10.1186/1472-6890-9-2 PMC2660363

[B35] Balabanova Y Drobniewski F Nikolayevskyy V Kruuner A Malomanova N Simak T Ilyina N Zakharova S Lebedeva N Alexander HL O’Brien R Sohn H Shakhmistova A Fedorin I An integrated approach to rapid diagnosis of tuberculosis and multidrug resistance using liquid culture and molecular methods in Russia PLoS One 2009 4 e7129 19774085 10.1371/journal.pone.0007129 PMC2744930

[B36] Dinnes J Deeks J Kunst H Gibson A Cummins E Waugh N Drobniewski F Lalvani A A systematic review of rapid diagnostic tests for the detection of tuberculosis infection Health Technol Assess 2007 11 1 196 17266837 10.3310/hta11030

[B37] Martin A Portaels F Palomino JC Colorimetric redox-indicator methods for the rapid detection of multidrug resistance in *Mycobacterium tuberculosis*: a systematic review and meta-analysis J Antimicrob Chemother 2007 59 175 183 17135182 10.1093/jac/dkl477

[B38] Moore DA Shah NS Alternative methods of diagnosing drug resistance - what can they do for me? J Infect Dis 2011 204 S1110 S1119 21996693 10.1093/infdis/jir448 PMC3192546

[B39] Leung E Minion J Benedetti A Pai M Menzies D Microcolony culture techniques for tuberculosis diagnosis: a systematic review Int J Tuberc Lung Dis 2012 16 16 23. i-iii 21986554 10.5588/ijtld.10.0065

[B40] Toit K Mitchell S Balabanova Y Evans CA Kummik T Nikolayevskyy V Drobniewski F The Colour Test for drug susceptibility testing of *Mycobacterium tuberculosis* strains Int J Tuberc Lung Dis 2012 16 1113 1118 22762424 10.5588/ijtld.11.0609

[B41] World Health Organization Non-commercial Culture and Drug Susceptibility Testing Methods for Screening Patients at Risk for Multidrug-Resistant Tuberculosis: Policy Statement. WHO/HTM/TB/2011.9 2011 Geneva, Switzerland: WHO 23586116

[B42] Baker LV Brown TJ Maxwell O Gibson AL Fang Z Yates MD Drobniewski FA Molecular analysis of isoniazid-resistant *Mycobacterium tuberculosis* isolates from England and Wales reveals the phylogenetic significance of the ahpC -46A polymorphism Antimicrob Agents Chemother 2005 49 1455 1464 15793126 10.1128/AAC.49.4.1455-1464.2005 PMC1068606

[B43] Telenti A Imboden P Marchesi F Lowrie D Cole S Colston MJ Matter L Schopfer K Bodmer T Detection of rifampicin-resistance mutations in *Mycobacterium tuberculosis* Lancet 1993 341 647 650 8095569 10.1016/0140-6736(93)90417-f

[B44] Takiff HE Salazar L Guerrero C Philipp W Huang WM Kreiswirth B Cole ST Jacobs WR Jr Telenti A Cloning and nucleotide sequence of *Mycobacterium tuberculosis* gyrA and gyrB genes and detection of quinolone resistance mutations Antimicrob Agents Chemother 1994 38 773 780 8031045 10.1128/aac.38.4.773 PMC284541

[B45] Bottger EC Pletschette M Andersson D Drug resistance and fitness in *Mycobacterium tuberculosis* infection J Infect Dis 2005 191 823 824 author reply 824 15688309 10.1086/427517

[B46] Sandgren A Strong M Muthukrishnan P Weiner BK Church GM Murray MB Tuberculosis drug resistance mutation database PLoS Med 2009 6 e2 19209951 10.1371/journal.pmed.1000002 PMC2637921

[B47] Drobniewski FA Diagnosing multidrug resistant tuberculosis in Britain Clinical suspicion should drive rapid diagnosis. BMJ 1998 317 1263 1264 10.1136/bmj.317.7168.1263 PMC1114199 9804707

[B48] Morgan M Kalantri S Flores L Pai M A commercial line probe assay for the rapid detection of rifampicin resistance in *Mycobacterium tuberculosis*: a systematic review and meta-analysis BMC Infect Dis 2005 5 62 16050959 10.1186/1471-2334-5-62 PMC1185540

[B49] Drobniewski F Nikolayevskyy V Balabanova Y Bang D Papaventsis D Diagnosis of tuberculosis and drug resistance: what can new tools bring us? Int J Tuberc Lung Dis 2012 16 860 870 22687497 10.5588/ijtld.12.0180

[B50] Ignatyeva O Kontsevaya I Kovalyov A Balabanova Y Nikolayevskyy V Toit K Dragan A Maxim D Mironova S Kummik T Muntean I Koshkarova E Drobniewski F Detection of resistance to second-line antituberculosis drugs using the Genotype® MTBDRsl assay: a multi-center evaluation and feasibility study J Clin Microbiol 2012 50 1593 1597 22378910 10.1128/JCM.00039-12 PMC3347138

[B51] Kontsevaya I Ignatyeva O Nikolayevskyy V Balabanova Y Kovalyov A Kritsky A Matskevich O Drobniewski F Diagnostic accuracy of the genotype MTBDRsl assay for rapid diagnosis of extensively drug-resistant tuberculosis in HIV-coinfected patients J Clin Microbiol 2013 51 243 248 23152552 10.1128/JCM.02513-12 PMC3536261

[B52] Vassall A van Kampen S Sohn H Michael JS John KR den Boon S Davis JL Whitelaw A Nicol MP Gler MT Khaliqov A Zamudio C Perkins MD Boehme CC Cobelens F Rapid diagnosis of tuberculosis with the Xpert MTB/RIF assay in high burden countries: a cost-effectiveness analysis PLoS Med 2011 8 e1001120 22087078 10.1371/journal.pmed.1001120 PMC3210757

[B53] World Health Organization Policy Statement: Automated Real-Time Nucleic Acid Amplification Technology for Rapid and Simultaneous Detection of Tuberculosis and Rifampicin Resistance: Xpert MTB/RIF system 2011 Geneva, Switzerland: WHO 2011 26158191

[B54] WHO monitoring of XPert MTB/RIF roll outhttp://www.who.int/tb/laboratory/mtbrifrollout/en/index.html

[B55] Pantoja A Fitzpatrick C Vassall A Weyer K Floyd K Xpert MTB/RIF for diagnosis of TB and drug-resistant TB: a cost and affordability analysis Eur Respir J 2012 [Epub ahead of print] 10.1183/09031936.00147912 23258774

[B56] Kirwan DE Cardenas MK Gilman RH Rapid implementation of new TB diagnostic tests: is it too soon for a global roll-out of Xpert MTB/RIF? Am J Trop Med Hyg 2012 87 197 201 22855746 10.4269/ajtmh.2012.12-0107 PMC3414551

[B57] Drobniewski FA Watterson SA Wilson SM Harris GS A clinical, microbiological and economic analysis of a national service for the rapid molecular diagnosis of tuberculosis and rifampicin resistance in *Mycobacterium tuberculosis* J Med Microbiol 2000 49 271 278 10707947 10.1099/0022-1317-49-3-271

[B58] White VL Moore-Gillon J Resource implications of patients with multidrug resistant tuberculosis Thorax 2000 55 962 963 11050268 10.1136/thorax.55.11.962 PMC1745633

[B59] Diel R Rutz S Castell S Schaberg T Tuberculosis: cost of illness in Germany Eur Respir J 2012 40 143 151 22267754 10.1183/09031936.00204611

[B60] Tuberculosis in the UK: annual report on tuberculosis surveillance in the UK, 2012 2012 London: Health Protection Agency http://www.hpa.org.uk/webc/HPAwebFile/HPAweb_C/1317134913404 accessed 14/08/2013

[B61] Keeler E Perkins MD Small P Hanson C Reed S Cunningham J Aledort JE Hillborne L Rafael ME Girosi F Dye C Reducing the global burden of tuberculosis: the contribution of improved diagnostics Nature 2006 444 49 57 17159894 10.1038/nature05446

[B62] Dowdy DW Chaisson RE Maartens G Corbett EL Dorman SE Impact of enhanced tuberculosis diagnosis in South Africa: a mathematical model of expanded culture and drug susceptibility testing Proc Natl Acad Sci USA 2008 105 11293 11298 18695217 10.1073/pnas.0800965105 PMC2516234

[B63] Abu-Raddad LJ Sabatelli L Achterberg JT Sugimoto JD Longini IM Jr Dye C Halloran ME Epidemiological benefits of more-effective tuberculosis vaccines, drugs, and diagnostics Proc Natl Acad Sci USA 2009 106 13980 13985 19666590 10.1073/pnas.0901720106 PMC2720405

[B64] Ruddy M Balabanova Y Graham C Fedorin I Malomanova N Elisarova E Kuznetznov S Gusarova G Zakharova S Melentyev A Krukova E Golishevskaya V Erokhin V Dorozhkova I Drobniewski F Rates of drug resistance and risk factor analysis in civilian and prison patients with tuberculosis in Samara Region, Russia Thorax 2005 60 130 135 15681501 10.1136/thx.2004.026922 PMC1747303

[B65] Balabanova Y Fedorin I Kuznetsov S Graham C Ruddy M Atun R Coker R Drobniewski F Antimicrobial prescribing patterns for respiratory diseases including tuberculosis in Russia: a possible role in drug resistance? J Antimicrob Chemother 2004 54 673 679 15317742 10.1093/jac/dkh383

[B66] Faustini A Hall AJ Perucci CA Risk factors for multidrug resistant tuberculosis in Europe: a systematic review Thorax 2006 61 158 163 16254056 10.1136/thx.2005.045963 PMC2104570

[B67] Silin M Laraque F Munsiff SS Crossa A Harris TG The impact of monitoring tuberculosis reporting delays in New York City J Public Health Manag Pract 2010 16 E09 17 20689383 10.1097/PHH.0b013e3181c87ae5

[B68] Paynter S Hayward A Wilkinson P Lozewicz S Coker R Patient and health service delays in initiating treatment for patients with pulmonary tuberculosis: retrospective cohort study Int J Tuberc Lung Dis 2004 8 180 185 15139446

[B69] Theron G Pinto L Peter J Mishra HK van Zyl-Smit R Sharma SK Dheda K The use of an automated quantitative polymerase chain reaction (Xpert MTB/RIF) to predict the sputum smear status of tuberculosis patients Clin Infect Dis 2012 54 384 388 22139854 10.1093/cid/cir824 PMC3258275

[B70] Turett GS Telzak EE Torian LV Blum S Alland D Weisfuse I Fazal BA Improved outcomes for patients with multidrug-resistant tuberculosis Clin Infect Dis 1995 21 1238 1244 8589149 10.1093/clinids/21.5.1238

[B71] Gandhi NR Moll A Sturm AW Pawinski R Govender T Lalloo U Zeller K Andrews J Friedland G Extensively drug-resistant tuberculosis as a cause of death in patients co-infected with tuberculosis and HIV in a rural area of South Africa Lancet 2006 368 1575 1580 17084757 10.1016/S0140-6736(06)69573-1

[B72] Balabanova Y Radiulyte B Davidaviciene E Hooper R Ignatyeva O Nikolayevskyy V Drobniewski FA Survival of drug resistant tuberculosis patients in Lithuania: retrospective national cohort study BMJ Open 2011 1 e000351 10.1136/bmjopen-2011-000351 PMC3225583 22123922

[B73] Drobniewski F Eltringham I Graham C Magee JG Smith EG Watt B A national study of clinical and laboratory factors affecting the survival of patients with multiple drug resistant tuberculosis in the UK Thorax 2002 57 810 816 12200527 10.1136/thorax.57.9.810 PMC1746427

[B74] Balabanova Y Nikolayevskyy V Ignatyeva O Kontsevaya I Rutterford CM Shakhmistova A Malomanova N Chinkova Y Mironova S Fedorin I Drobniewski FA Survival of civilian and prisoner drug-sensitive, multi- and extensive drug- resistant tuberculosis cohorts prospectively followed in Russia PLoS One 2011 6 e20531 21695213 10.1371/journal.pone.0020531 PMC3112205

[B75] Shah M DiPietro D Greenbaum A Ketemepi S Martins-Evora M Marsiglia V Dorman SE Programmatic impact of QuantiFERON-TB Gold In-Tube implementation on latent tuberculosis diagnosis and treatment in a public health clinic PLoS One 2012 7 e36551 22586476 10.1371/journal.pone.0036551 PMC3346719

[B76] Pai M Kalantri S Dheda K New tools and emerging technologies for the diagnosis of tuberculosis: part I. Latent tuberculosis Expert Rev Mol Diagn 2006 6 413 422 16706743 10.1586/14737159.6.3.413

[B77] Drobniewski F Balabanova Y Zakamova E Nikolayevskyy V Fedorin I Rates of latent tuberculosis in health care staff in Russia PLoS Med 2007 4 e55 17298167 10.1371/journal.pmed.0040055 PMC1796908

[B78] Supply P Allix C Lesjean S Cardoso-Oelemann M Rüsch-Gerdes S Willery E Savine E de Haas P van Deutekom H Roring S Bifani P Kurepina N Kreiswirth B Sola C Rastogi N Vatin V Gutierrez MC Fauville M Niemann S Skuce R Kremer K Locht C van Soolingen D Proposal for standardization of optimized mycobacterial interspersed repetitive unit-variable-number tandem repeat typing of Mycobacterium tuberculosis J Clin Microbiol 2006 44 4498 4510 17005759 10.1128/JCM.01392-06 PMC1698431

[B79] Roetzer A Diel R Kohl TA Rückert C Nübel U Blom J Wirth T Jaenicke S Schuback S Rüsch-Gerdes S Supply P Kalinowski J Niemann S Whole genome sequencing versus traditional genotyping for investigation of a *Mycobacterium tuberculosis* outbreak: a longitudinal molecular epidemiological study PLoS Med 2013 10 e1001387 23424287 10.1371/journal.pmed.1001387 PMC3570532

[B80] Cattamanchi A Hopewell PC Gonzalez LC Osmond DH Masae Kawamura L Daley CL Jasmer RM A 13-year molecular epidemiological analysis of tuberculosis in San Francisco Int J Tuberc Lung Dis 2006 10 297 304 16562710

[B81] Kato-Maeda M Metcalfe JZ Flores L Genotyping of *Mycobacterium tuberculosis*: application in epidemiologic studies Future Microbiol 2011 6 203 216 21366420 10.2217/fmb.10.165 PMC4296029

[B82] Jasmer RM Hahn JA Small PM Daley CL Behr MA Moss AR Creasman JM Schecter GF Paz EA Hopewell PC A molecular epidemiologic analysis of tuberculosis trends in San Francisco, 1991–1997 Ann Intern Med 1999 130 971 978 10383367 10.7326/0003-4819-130-12-199906150-00004

[B83] Walker TM Ip CL Harrell RH Evans JT Kapatai G Dedicoat MJ Eyre DW Wilson DJ Hawkey PM Crook DW Parkhill J Harris D Walker AS Bowden R Monk P Smith EG Peto TE Whole-genome sequencing to delineate *Mycobacterium tuberculosis* outbreaks: a retrospective observational study Lancet Infect Dis 2013 13 137 146 23158499 10.1016/S1473-3099(12)70277-3 PMC3556524

[B84] Bryant JM Schürch AC van Deutekom H Harris SR de Beer JL de Jager V Kremer K van Hijum SA Siezen RJ Borgdorff M Bentley SD Parkhill J van Soolingen D Inferring patient to patient transmission of *Mycobacterium tuberculosis* from whole genome sequencing data BMC Infect Dis 2013 13 110 23446317 10.1186/1471-2334-13-110 PMC3599118

